# Ultimate and Deflection Performance of Concrete Beams Strengthened in Flexure with Basalt-Textile-Reinforced Polymer Mortar

**DOI:** 10.3390/polym15020445

**Published:** 2023-01-14

**Authors:** Jun Deng, Minting Zhong, Zhuojian Zhang, Miaochang Zhu

**Affiliations:** 1 School of Civil Engineering, Guangzhou University, Guangzhou 510006, China; 2 School of Civil and Transportation Engineering, Guangdong University of Technology, Guangzhou 510006, China

**Keywords:** basalt textile, BTRM, flexural strengthening, flexural strength, effective moment of inertia

## Abstract

This paper presents an investigation into the ultimate and serviceability behavior of concrete beams strengthened in flexure with basalt-textile-reinforced polymer mortar (BTRM). The bond performance at the interface between the BTRM and concrete was studied by performing single shear tests, and the effectiveness of using an adhesion promoter and impregnated resin for bond enhancement was explored. The results suggested that using an adhesion promoter and impregnated resin can improve the interfacial stress transfer and ensure the tensile failure of the basalt textile in BTRM. Afterward, four-point bending tests were conducted to study the flexural performance of strengthened beams. It was found that the flexural strength of strengthened beams increased with the amount of textile, and the strength increase was more prominent for the strengthened beams with end anchorages. The increase in the failure force was up to 37% for the beam strengthened with five layers of the textile and an end anchorage. The calculated flexural strength exhibited a percentage error of no more than 7% compared to the test results. In addition, the Bischoff-I Equation can closely estimate the effective moment of inertia and provide an accurate prediction of deflection for strengthened beams.

## 1. Introduction

Fiber-reinforced polymers (FRPs) externally attached to concrete have been developed and are widely utilized for retrofitting and strengthening concrete beams [1,2,3,4]. The recognized merits of using FRPs are their light weight, high strength, good resistance to corrosion, and ease of installation. FRPs are made of an organic matrix and require the use of a bonding agent to be attached to the concrete surface. The matrix and bonding agent are usually made of different types of resins, of which epoxy resin is the most extensively used. Currently, the bond behavior [5,6] between the externally bonded FRP and concrete and the effectiveness of the corresponding flexural strengthening systems [7] have been effectively addressed. However, problems arising from using epoxy resin have been identified [8,9], such as the low glass transition temperature, limitations to cold and/or wet concrete surfaces, poor long-term durability, and chemical toxicity induced by environmental and health issues.

The advent of cement-based composites made by replacing the organic matrix with inorganic cementitious materials represents an attractive and prospective alternative that allows the above problems to be surmounted. In addition, using cementitious materials can provide the protection of fiber reinforcement, good resistance to temperature, and sound compatibility with the concrete substrate. Such composites are called textile-reinforced mortar (TRM), and different acronyms, such as TRC and FRCM, were used in different studies [10,11,12,13,14,15]. Various types of fibers have been exploited for making TRM [16,17,18,19,20], including carbon, PBO (poliparafenilenbenzobisoxazole), glass, aramid, and basalt. 

Basalt fibers are made of melted basalt minerals drawn through a platinum–rhodium alloy wiredrawing leaky plate and are recognized as green, eco-friendly, high-performance synthetic fibers. Basalt fibers show good mechanical properties and durability at a highly competitive cost. 

Experimental investigations have been conducted on basalt-textile-reinforced mortar, revealing that BTRM shows good mechanical properties. The factors that affect the mechanical performance of BTRM have been distinguished, including the reinforcement amount [21], the mortar type [21,22] and the addition of different chopped fibers [23,24], and the effects of these factors have been addressed. Except for clarifying the mechanical properties of BTRM itself, limited studies are available regarding the effectiveness of BTRM on concrete beams strengthened in flexure [25,26] compared to TRM strengthening with other textiles [27,28]. These limited studies mainly focused on the failure mode and moment capacity. The existing studies [27,28] discovered that the primary failure of strengthened beams often occurred by debonding at interfaces such as TRM–concrete interfaces and textile–matrix interfaces. The occurrence of debonding reduces the strength exploitation of basalt fibers and limits the effectiveness of the strengthening systems. Preventing debonding remains a critical issue to be solved.

In addition to the ultimate behavior, the strengthened concrete members should carry enough stiffness to resist deformation. Serviceability represents a crucial condition that should be satisfied for ordinary members. However, no investigations are available regarding the deflection behavior of BTRM-strengthened beams. Thus, the accurate calculation of member deflection cannot be performed at present, which constituted a motivation for the present study.

This study intended to investigate the flexural performance of concrete beams strengthened in flexure with BTRM in terms of the ultimate and deflection behavior. Single shear tests were first conducted to explore the failure mechanism and stress transfer between the BTRM and concrete and determine the effectiveness of using an adhesion promoter and impregnated resin. Afterward, four-point bending was applied to strengthened concrete beams with various BTRM amounts to study the flexural performance, mainly in terms of failure, load-deflection behavior, and ultimate flexural strength as well as the effective moment of inertia. The flexural strength was calculated by a sectional analysis and compared with experimental results to determine the prediction accuracy. Additionally, the effective moment of inertia determined from the load-deflection response of strengthened beams was compared with existing equations to determine the optimal Equation with the best accuracy.

## 2. Experimental Program

The experimental program included single shear tests and bending tests. The single shear tests were conducted on BTRM–concrete interface specimens to study the bond properties between the BTRM and concrete to determine the effectiveness of using an adhesion promoter and impregnated resin and obtain the bond length required to achieve the tensile capacity of basalt textile. The bending tests were performed to study the flexural performance of concrete beams strengthened with BTRM in terms of failure modes, moment capacity, and deflection behavior, verifying the effectiveness of BTRM in improving the ultimate and deflection behavior of concrete beams. 

### 2.1. Single Shear Tests

#### 2.1.1. Materials and Specimens

The basalt textile used was a commercially available bidirectional coated textile with a mesh size of 25 mm × 25 mm. The elastic modulus and tensile strength of the basalt textile were 80 GPa and 1500 MPa, respectively. The primer and PCM were provided by the same supplier. The primer consisted of acrylic acid and water with a mix ratio, in weight, of 1:1. The PCM that incorporated polyvinyl alcohol fibers with a volume fraction of 0.1% was used as a matrix to make BTRM and the bonding agent. The impregnation polymer was a two-component ambient-cured epoxy resin, and the mix ratio, in weight, between the base and hardener was 2:1. The flexural and compressive strengths of PCM after curing for seven days were 10.0 MPa (COV = 0.051) and 55.6 MPa (COV = 0.058), respectively. Ready-mix concrete was used to fabricate BTRM–concrete interface specimens, and the 28-day compressive strength of 40.2 MPa (COV = 0.064) was determined based on compression tests on three cubic specimens. 

A total of nine BTRM–concrete interface specimens were used in single shear tests. An adhesion promoter was intended to enhance the adhesion between the PCM and concrete, and impregnated resin was intended to increase the strength utilization of the basalt textile. Three scenarios were considered, depending on whether the adhesion promoter and impregnated resin were used: (i) S0 specimens with neither an adhesion promoter nor impregnated resin; (ii) SP specimens with an adhesion promoter but no impregnated resin; and (iii) SPR specimens with both an adhesion promoter and impregnated resin. Three parallel specimens were tested for each scenario. 

**Figure 1:**
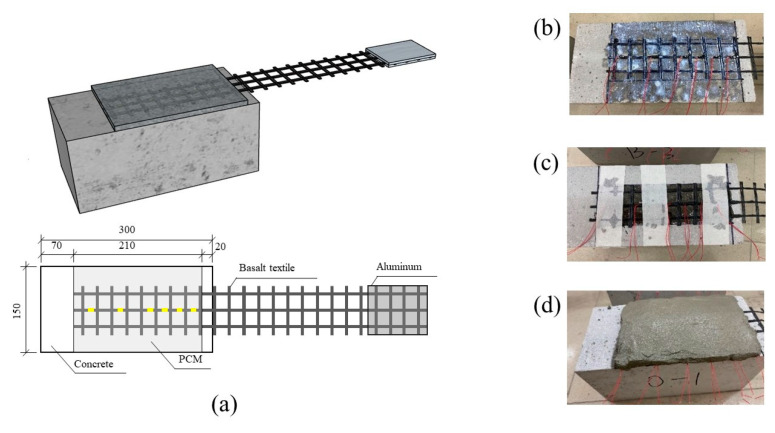
BTRM–concrete interface specimen arrangement (**a**) and preparation, including spreading adhesion promoter (**b**), applying impregnated resin (**c**), and covering PCM (**d**).

The BTRM–concrete interface specimens comprised one concrete prism with dimensions of 150 mm × 150 mm × 300 mm and the externally bonded BTRM. Figure 1 presents the specimen arrangement and geometries for single shear tests. Prior to the attachment of BTRM, the bottom casting surfaces of the concrete prisms were ground to expose coarse aggregate and wiped using alcohol. Subsequently, the basalt textile was positioned on the treated concrete surface and covered with PCM for the S0 specimens. Regarding the SP specimens, an adhesion promoter was spread on the concrete surface and basalt textile, followed by applying PCM. Note that the adhesion promoter was utilized appropriately so that the concrete was in a surface-saturated condition with no visible excessive liquid. The SPR specimens were fabricated by first using impregnated resin to attach the basalt textile on the concrete surface, spreading an adhesion promoter after curing the resin for 24 h, and applying PCM. The thickness of the PCM layer was 10 mm for all specimens. To avoid local shear failure near the concrete edges, unbonded areas measuring 20 mm × 150 mm were left at the ends of the concrete prisms.

#### 2.1.2. Test Implementation

**Figure 2:**
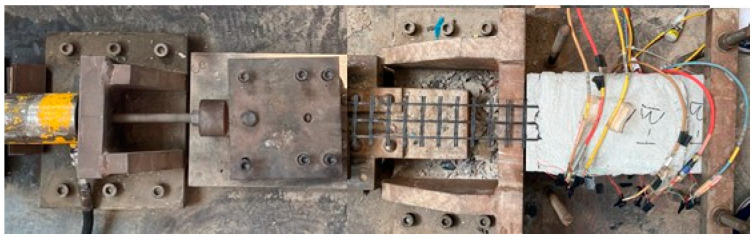
Test setup for single shear tests.

Figure 2 shows the test setup for single shear tests. The BTRM–concrete interface specimens were affixed to a steel rig and gripped by two bolted steel clamps connected to a hydraulic jack. The single shear tests were performed in load-controlled mode at 1 kN/min. The external load applied to the textile was consistent with the longitudinal axis of the textile, which was measured using a loading cell. Aluminum plates were bonded onto the end of the basalt textile with an epoxy resin to facilitate gripping. Six electric strain gauges mounted along the basalt textile were used to obtain variations in the textile strain during loading, as shown in Figure 1. 

### 2.2. Bending Tests 

#### 2.2.1. Materials and Concrete Beams

**Figure 3:**
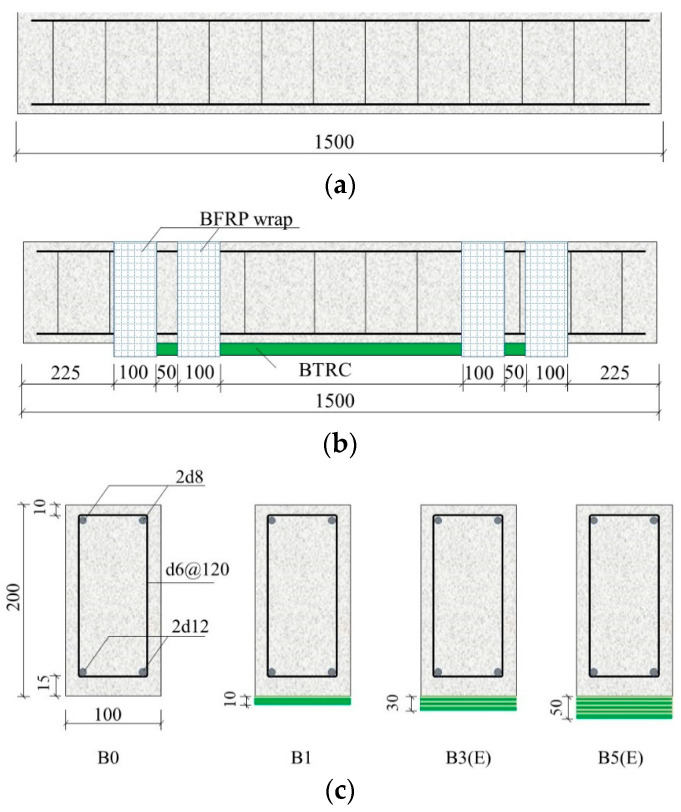
Specimen arrangement for control and strengthened beams. (**a**) Control beam. (**b**) BTRM-strengthened beam. (**c**) Sectional details.

Six concrete beams with dimensions of 100 mm × 200 mm × 1500 mm were fabricated in the present study. Table 1 shows the specimen arrangement for the bending tests. All beams were configured with two 10 mm diameter steel bars as tension reinforcement and two 8 mm diameter steel bars in the compression zone to support steel stirrups, as shown in Figure 3. The longitudinal tension and support reinforcements were HRB400-grade. HPB235-grade two-legged steel stirrups were center-to-center spaced at 120 mm along the beam length. This steel configuration ensured that flexural failure would occur as the governing mode. 

One of the concrete beams was used as a control beam without strengthening and was labeled as B0. Other beams were strengthened with BTRM incorporated into one layer or multiple layers of the basalt textile. Strengthened beams were identified as B*x*, where *x* indicates the number of textile layers, as shown in Table 1. To delay or avoid the premature failure induced by the debonding, end anchorages were used near the terminal of the BTRM by wrapping the whole section with a basalt fiber sheet, as shown in Figure 3. If end anchorages existed, the beam was indicated by adding the suffix E. For instance, B5E distinguishes a beam strengthened with five basalt textiles layers and end anchorages. 

The basalt textile and PCM for strengthening concrete beams were the same as those for the interface specimens. The concrete beams were cast with another batch of concrete with a compressive strength of 34.8 MPa based on compression test results. The yielding and ultimate tensile strengths of HRB400 steel bars were 450 Mpa and 610 Mpa, respectively, and the corresponding strengths were 235 Mpa and 250 Mpa for HPB235 steel stirrups. The elastic modulus for the longitudinal steel reinforcement and stirrups was 200 Gpa. The elastic modulus and tensile strength of the basalt fiber sheet were 105 Gpa and 2131 Mpa, respectively. 

The application of BTRM followed the same procedure for fabricating BTRM–concrete interface specimens. The depth between two adjacent layers of basalt textile was 10 mm, resulting in total thicknesses of 20 mm and 40 mm for BTRM with three and five layers of the textile. If the end anchorage existed, the PCM surface was subjected to the same treatment as concrete, and the basalt fiber sheet was used to wrap the beam section with the impregnated resin. 

#### 2.2.2. Test Implementation

**Figure 4:**
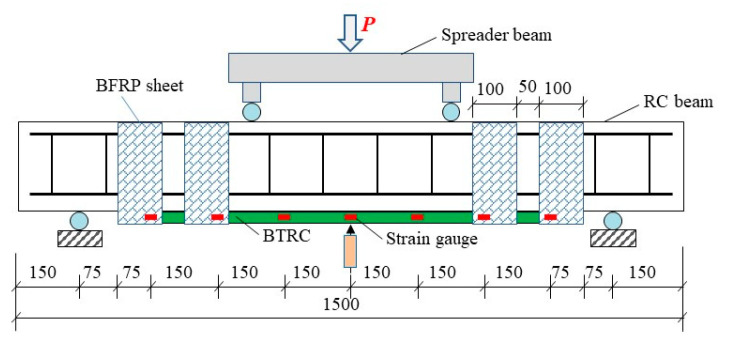
Test setup for bending tests.

All beams were tested under four-point bending with a simply supported condition, as shown in Figure 4. The span length between the two supports was 1200 mm, and the distance between the two loading points was 450 mm. The bending tests were performed in load-controlled mode. The external force was generated using a hydraulic jack and was distributed equally to loading points through a steel spreader beam. The force was applied with a step length of 5 kN at a rate of 0.033 kN/s before concrete cracking occurred. Then, the step length and the rate changed to 10 kN and 0.067 kN/s. The applied force was maintained for 300 s at the end of each loading step. A loading cell equipped with the jack was used to measure the applied force. The deflection at the midspan was measured using a linear variable differential transformer. In addition, seven strain gauges were attached to the basalt textile to measure the strain in the textile along the longitudinal axis.

## 3. Test Results

### 3.1. Single Shear Test Results

#### 3.1.1. Failure Mode

**Figure 5:**
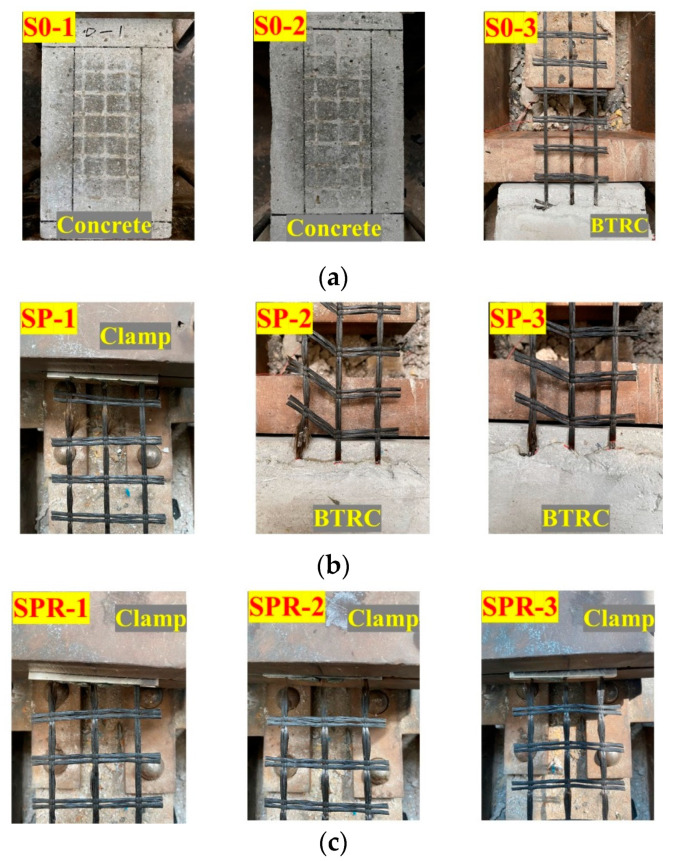
Failure modes observed in single shear tests. (**a**) S0. (**b**) SP. (**c**) SPR.

Various failure modes were observed in the single shear tests, as shown in Figure 5. S0-1 and S0-2 exhibited the detachment of BTRM from the concrete–PCM interface, and S0-3 failed due to significant slippage of the textile along the interface, which indicated the weakness of the adhesion between the concrete and PCM and the necessity for adhesion enhancement. Regarding SP2 and SP3, the failure occurred due to the tensile failure of the basalt textile near the loading end of the bonded area, while the failure developed near the clamps for SP1. All SPR specimens showed a failure mode due to the tensile fracture of the basalt textile near the clamps. Therefore, the use of an adhesion promoter and impregnated resin improved the interfacial adhesion, as indicated by the changes in the failure mode.

#### 3.1.2. Maximum Applied Force

**Figure 6:**
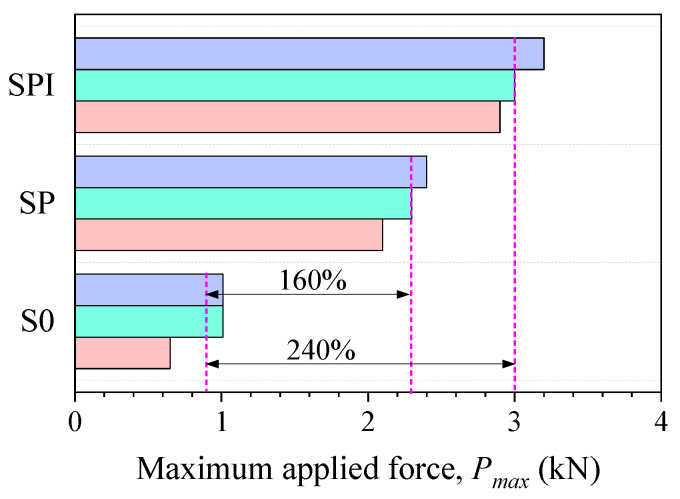
The maximum applied force measured in single shear tests.

Figure 6 presents the maximum applied force obtained in single shear tests. The average maximum applied force was 0.88 kN for the S0 specimens, and an increase of 160% was observed for the SP specimens. The increase was due to the beneficial effect of the adhesion promoter in enhancing interfacial adhesion, which resulted in the failure mode shifting from interfacial debonding to textile failure. If the adhesion promoter and impregnated resin were applied to SPR specimens, an additional increase of 80% in the maximum applied force was noticed compared to the SP specimens. Such an additional increase can be attributed to the consolidating effect of the impregnated resin on the basalt textile, which increased the textile tensile strength.

#### 3.1.3. Strains in Basalt Textile

**Figure 7:**
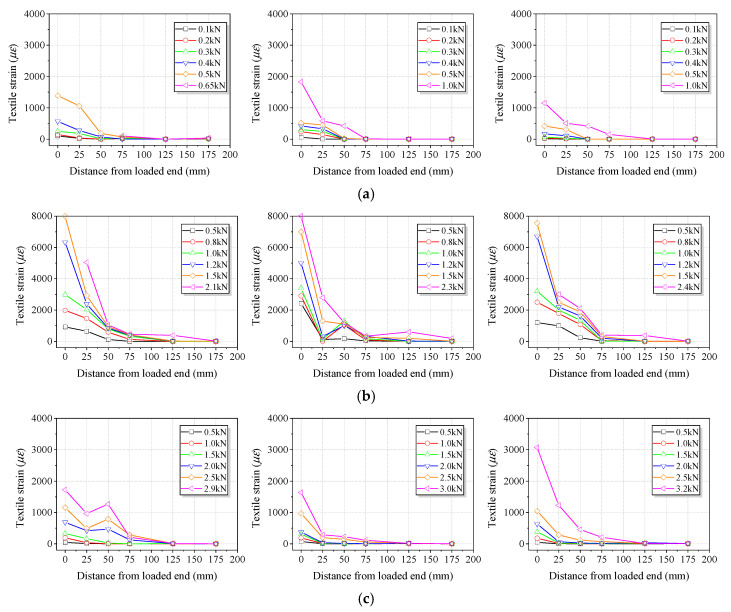
Basalt textile strain profiles along the bond length obtained from single shear tests. (**a**) S0. (**b**) SP. (**c**) SPI.

The strain profiles of basalt textile are plotted versus the distance from the loaded end at different loads for BTRM–concrete specimens in Figure 7. All the BTRM–concrete specimens exhibited a similar overall tendency where the textile strain decreased as the distance away from the loaded end increased. In addition, by increasing the applied force, the increase in the textile strain could be registered at different locations. The differences in the maximum measured strain were evident for parallel specimens, probably due to variations in material properties and specimen preparation and possible bending effects of the textile. Non-uniform load distribution among different strands of the textile occurred, indicated by the asynchronous failure of the strands. In addition, the maximum textile strain was lower for SPR specimens than for SP specimens, which was inconsistent with the maximum applied force findings. The reason was that the impregnated resin stiffened the textile and significantly increased the interlock action offered by orthogonal strands. Since the strain gauge was attached behind the first transverse strand within the bonded area, the influence of the interlock action could not be captured. 

The analysis of the textile strain profiles also aimed to determine the critical bond length required to achieve the tensile strength of the textile. As the failure of the S0 specimens did not occur from the tensile failure of the textile, the critical bond length could not be determined. According to Figure 7b,c, the critical bond length could be taken as 175 mm and 125 mm for the SP and SPR specimens, respectively. The shorter critical bond length for SPR specimens also indicated that the impregnated resin could enhance the effectiveness of the force transition from the textile to concrete. Therefore, the adhesion promoter and impregnated resin were used when preparing the strengthened concrete beams.

### 3.2. Four-Point Bending Test Results

#### 3.2.1. Failure Mode

**Figure 8:**
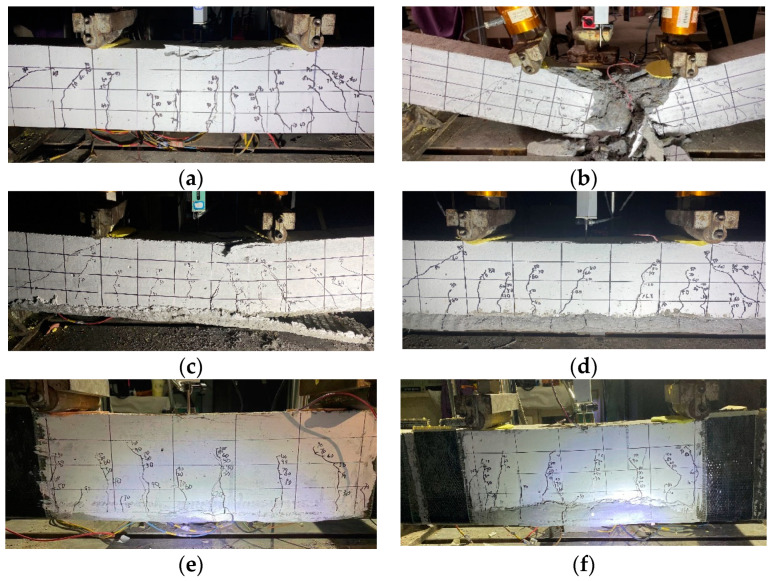
Failure modes of control and strengthened concrete beams. (**a**) B0. (**b**) B-1. (**c**) B-3. (**d**) B-5. (**e**) B-3-E. (**f**) B-5-E.

Figure 8 shows the failure modes for the tested beams. As expected, B0 exhibited a typical flexural failure pattern due to the crushing of concrete in the compression zone at the midspan. The failure of B1 also occurred due to concrete crushing following the tensile fracture of the basalt textile. Abrupt failure was observed for B1 due to the beam being tested under a load-controlled condition. For B3 and B5, the failure was initiated by the end peeling of BTRM at the interface between the textile and mortar. With a further increment in the applied force, concrete crushing at the compression face occurred for B3, along with the tensile failure of part of the textile layer. As for B5, one inclined crack developed outside the constant moment region and quickly grew toward the nearest loading points, leading to flexural failure preceded by shear failure. By wrapping the beam section using a basalt fiber sheet, adequate end anchorage was achieved so that the end debonding of BTRM would not occur. The shear capacity of strengthened beams could be increased as well to ensure the occurrence of flexural failure. Therefore, B3E and B5E experienced typical flexural failure.

#### 3.2.2. Load-Deflection Behavior

**Figure 9:**
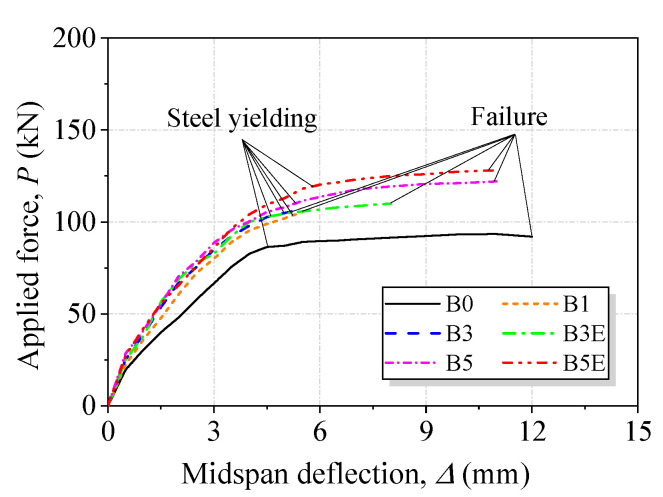
Applied force versus midspan deflection curves for tested beams.

The applied force was plotted against the midspan deflection for the tested beams, as shown in Figure 9. All beams exhibited a similar overall trend featuring three distinct phases. Little difference could be observed in the beams in the first phase, where the section was not cracked. The first bend point of the load-deflection curves was taken as the cracking load. The deflection of the beams was minimal before cracking. The first crack appeared at a deflection of no more than 0.8 mm. In the second phase (post-cracking but not steel yielding), the strengthened beams experienced stiffened deflection behavior compared to B0. After steel yielding was initiated, the load-deflection curves experienced the last phase, where a sudden increase in the deflection occurred. With the use of end anchorage, B3E exhibited a greater deflection capacity than B3. On the contrary, the effect of end anchorage on the ultimate deflection of the beams with five textile layers was negligible, while an overall upward shift in the load-deflection curve was observed.

#### 3.2.3. Maximum Applied Force

**Figure 10:**
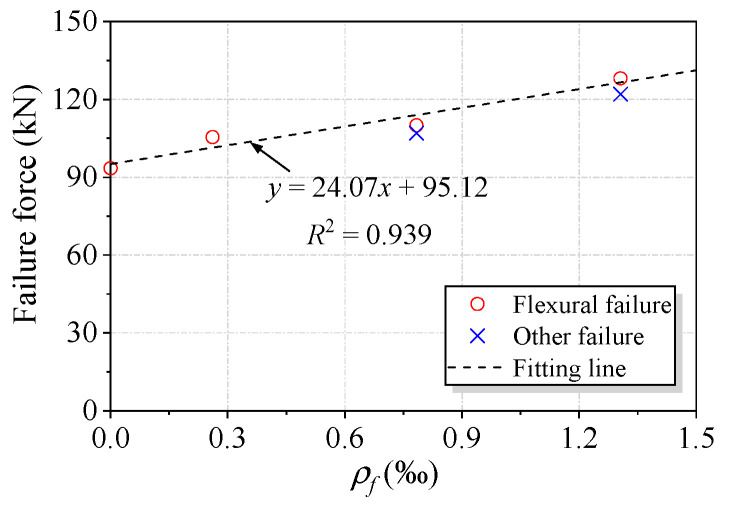
Relationship between the failure force and the textile reinforcement amount for the tested beams.

The maximum applied force, defined as the failure force (*P_u_)*, was measured during bending tests and is presented in Table 1. B0 showed a *P_u_* value of 93.5 kN. For the strengthened beams, *P_u_* ranged between 105.5 kN for B1 and 128.1 kN for B5E. The increases were 13% and 37% for B1 and B5E, respectively, compared to B0. In addition, the difference in *P_u_* between B3 and B3E was 4%, and this difference was 6% between B5 and B5E. Although end anchorage had a beneficial effect on the deflection and failure mode, slight differences in *P_u_* appeared for the strengthened beams with and without end anchorage. In addition, *P_u_* increased with an increase in the reinforcement amount of the textile, and a linear dependence was found between *P_u_* and the textile reinforcement amount, as shown in Figure 10.

#### 3.2.4. Strains in Basalt Textile

**Figure 11:**
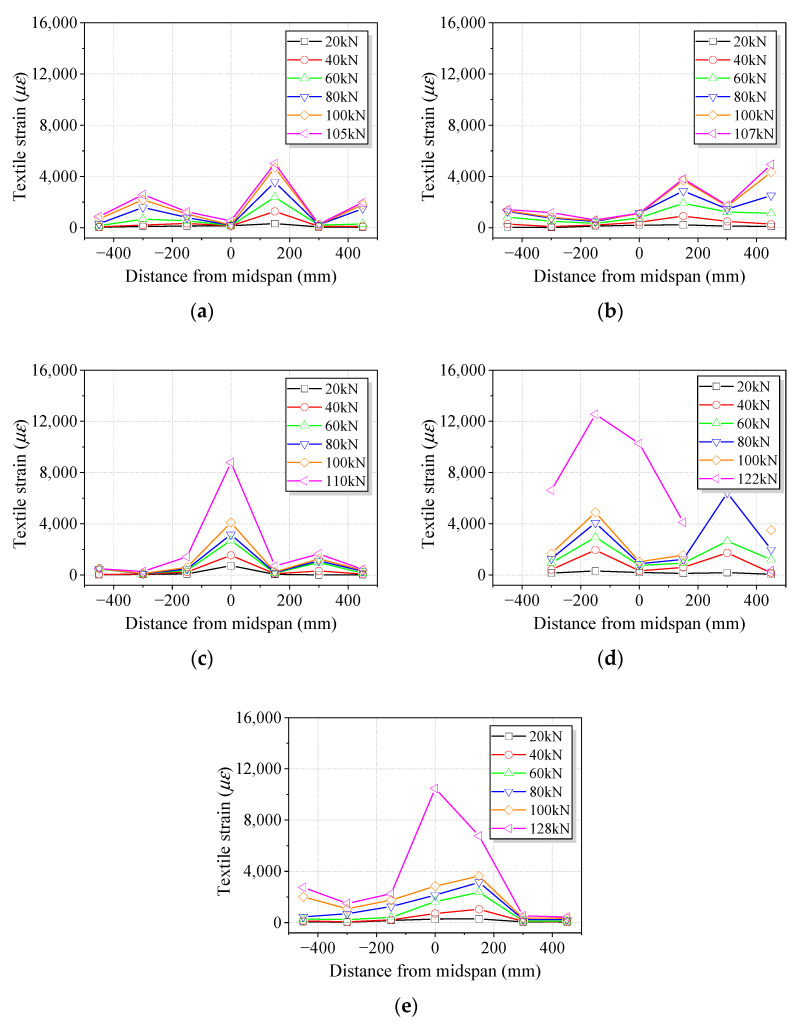
Strain profiles of basalt textile for strengthened beams. (**a**) B1. (**b**) B3. (**c**) B3E. (**d**) B5. (**e**) B5E.

Figure 11 shows the strains in basalt textile for strengthened beams. The textile strain culminated near the midspan within the constant moment region and diminished as the distance from the midspan increased for small applied forces. When increasing the applied force, the textile strain at one end rose quickly, exceeded the midspan strain for B3 and B5, and eventually led to the development of end debonding, as shown in Figure 8. The strain gauge at the end of the textile stopped reading after 100 kN for B5. If the end anchorage was installed, the textile strain diminished at the end, indicating the beneficial effects of the end anchorage on reducing stress concentration and the effectiveness in preventing end debonding. In addition, the exact position where the maximum textile strain occurred was not easy to identify due to the random cracking process of concrete at the tension face. Therefore, the registered maximum textile strain was different, although similar tensile failures of textile could be observed in B1, B3E, and B5E.

## 4. Discussion

### 4.1. Ultimate Moment Calculation

The ultimate moment should be determined based on the governing failure mode. The present study addressed the ultimate moment calculation corresponding to the flexural failure for concrete beams. A typical sectional analysis was performed for the BTRM-strengthened beams, accompanied by the following widely accepted assumptions: (1) planar sections remain planes after cracking; (2) the contribution of concrete in tension on the moment capacity is negligible; (3) steel reinforcement has an elastic–plastic behavior, and the basalt textile shows a linear behavior up to failure; and (4) perfects bond between different materials exist.

A rectangular stress block approach given by ACI 318-19, developed for ordinary reinforced concrete beams, was used to analyze the ultimate moment of concrete beams strengthened with BTRM. According to the ACI 318-19 code, the equivalent stress magnitude and the position of the resultant compression force of the block could be determined using Equations (1) and (2):

(1) $$\beta_{1}=\frac{4\varepsilon_{c}^{\prime }-\varepsilon_{ct}}{6\varepsilon_{c}^{\prime }-2\varepsilon_{ct}}$$

(2) $$\alpha_{1}=\frac{1}{\beta_{1}}\left[\left(\frac{\varepsilon_{ct}}{\varepsilon_{c}^{\prime }}\right)-\frac{1}{3}{\left(\frac{\varepsilon_{ct}}{\varepsilon_{c}^{\prime }}\right)}^{2}\right]$$

where *β*_1_ represents the ratio of the stress block height to the neutral axis depth of the cracked section, and *α*_1_ indicates the ratio of the block stress to the compressive strength of concrete. Both *α*_1_ and *β*_1_ are related to the concrete strain corresponding to the compressive strength ($\varepsilon_{c}^{\prime }$) and the actual strain at the top compression fiber (*ε_c__t_)*. Once flexural failure occurs, *ε_ct_* reaches the ultimate compressive strain for concrete (0.0035). Based on the strain compatibility, the strains in the compression steel, tension steel, and textile can be obtained as follows:

(3) $$\varepsilon_{s}^{\prime }=\frac{d_{s}^{\prime }}{x_{c}}\varepsilon_{ct}$$

(4) $$\varepsilon_{s}=\frac{d_{s}}{x_{c}}\varepsilon_{ct}$$

(5) $$\varepsilon_{f}=\frac{d_{t}-x_{c}}{x_{c}}\varepsilon_{ct}$$

where $\varepsilon_{s}^{\prime }$, *ε_s_,* and *ε_f_* represent the strains in the compression steel, tension steel, and textile; *x_c_* is the depth of the neutral axis of the cracked section; and $d_{s}^{\prime }$, *d_s_,* and *d_f_* indicate the depth of the compression steel, tension steel, and textile into the extreme compression fiber, respectively.

Considering the force equilibrium about the axis normal to the section, Equation (6) can be established. By inserting Equations (3)–(5), *x_c_* can be determined; therefore, the strains in different materials can be obtained. Afterward, the ultimate moment (*M_u_)* can be calculated by summing the moment about the neutral axis for different components, as expressed in Equation (7).

(6) $$\alpha_{1}f_{c}b\beta_{1}+A_{s}^{\prime }E_{s}\varepsilon_{s}^{\prime }=A_{s}E_{s}\varepsilon_{s}+A_{f}E_{f}\varepsilon_{f}$$

(7) $$M_{u}=\alpha_{1}f_{c}b\beta_{1}x_{c}\left(x_{c}-\frac{\beta_{1}x_{c}}{2}\right)+A_{s}^{\prime }E_{s}\varepsilon_{s}^{\prime }\left(x_{c}-d_{s}^{\prime }\right)+A_{s}E_{s}\varepsilon_{s}\left(d_{s}-x_{c}\right)+A_{f}E_{f}\varepsilon_{f}\left(d_{f}-x_{c}\right)$$

The ultimate moments determined from the maximum applied force were compared with the calculated values for the beams. An inspection of Table 2 revealed that the differences between the experimental and calculated *M_u_* values were no more than 7%, and the overall ratio between them was 0.96, indicating good accuracy of the moment capacity prediction based on the ACI code. To achieve more statistically significant conclusions, further studies are needed to increase the number of tests of the flexural strengthening of concrete beams with BTRM.

### 4.2. Deflection Behavior 

Flexural members should carry enough stiffness to resist a deflection that may affect the safety and serviceability conditions. Therefore, it is necessary to check the deflection of strengthened members to satisfy the serviceability requirements. The deflection behavior of concrete beams can be computed using an elastic deflection equation that incorporates the elastic modulus of concrete (*E_c_)* and the effective moment of inertia (*I_e_)* for any specific applied moment (*M_a_)*. *I_e_* is used to model the nonlinear behavior of members after concrete cracking and reflects the gradual transition of member stiffness from the uncracked section to a completely cracked section. The value of *I_e_* can be experimentally evaluated for concrete members based on the relationship between the applied force and deflection, which is contingent on the boundary condition and the type of loading. The equation to calculate *I_e_* is formulated in Equation (8) for simply supported beams under four-point bending.

(8) $$I_{e-Exp}=\frac{Pa\left(3L^{2}-4a^{2}\right)}{48E_{c}\Delta }$$

where *P* is the applied force; *L* and *a* are the beam span and shear span; *E_c_* is the elastic modulus of concrete, which can be calculated as per ACI 318-19; and Δ is the midspan deflection.

#### 4.2.1. Existing Prediction Equations of *I_e_*

The prediction equation of *I_e_* was originally proposed by Branson for steel-reinforced concrete. The Branson equation takes the actual member stiffness as a weighted average of the uncracked (*E_c_I_un_*) and cracked (*E_c_I_cr_*) stiffness. Modifications have been made to the Branson equation to improve the prediction accuracy by accounting for different factors that affect the member stiffness. These factors include the tensile strength and stiffness of concrete, the amount and type of reinforcement, shrinkage, and creep. For example, Gao et al. proposed a modified version, adopted using the ACI 440 code [29], of the Branson equation for FRP-bar-reinforced concrete, as shown in Equation (9). This equation was obtained by considering the reduced tension stiffening due to the lower elastic modulus of FRP bars.

(9) $$I_{e-ACI\;440}={\left(\frac{M_{cr}}{M_{a}}\right)}^{3}\beta_{d}I_{un}+\left[1-{\left(\frac{M_{cr}}{M_{a}}\right)}^{3}\right]I_{cr}$$

where *M_cr_* and *M_a_* are the cracking moment and the applied moment of interest and *β_d_* is a correction factor that was empirically derived for FRP-bar-reinforced concrete. 

For a glass FRP bar, Alsayed et al. [30] developed another modified equation (Equation (10)) for computing the *I_e_* of reinforced concrete beams. The difference between the equation of Alsayed et al. and the ACI 440 Equation is that the power changed to 5.5.

(10) $$I_{e-\mathrm{Alsayed}}={\left(\frac{M_{cr}}{M_{a}}\right)}^{5.5}\beta_{d}I_{un}+\left[1-{\left(\frac{M_{cr}}{M_{a}}\right)}^{5.5}\right]I_{cr}$$

By addressing the limitation of the Branson Equation, which may not give an accurate estimate of *I_e_* for an *I_un_/I_cr_* ratio larger than 4.0, Bischoff [31,32] developed a simple yet rational equation (Equation (11), referred to as Bischoff-I) to compute *I_e_*, regardless of the amount or type of reinforcement (steel or FRP). It was found that taking a weighted average of flexibility (the reciprocal of stiffness) is more appropriate to model deflection behavior over a wide range of member stiffness values. In some cases, the member stiffness is underestimated using the uniform value of *I_e_* based on the applied moment at the critical section, and an overprediction of deflection is the result. To account for variations in stiffness along the span, an additional integration factor, *γ,* that is dependent on the boundary condition and the loading type was proposed [33], as shown in Equation (12) (referred to as Bischoff-II). For simply supported beams under four-point bending, *γ* can be determined using Equation (12a).

(11) $$I_{e-Bischoff-I}=\frac{I_{cr}}{1-\left(1-\frac{I_{cr}}{I_{un}}\right){\left(\frac{M_{cr}}{M_{a}}\right)}^{2}}$$

(12) $$I_{e-Bischoff-II}=\frac{I_{cr}}{1-\gamma \left(1-\frac{I_{cr}}{I_{un}}\right){\left(\frac{M_{cr}}{M_{a}}\right)}^{2}}$$

(12a) $$\gamma =\frac{3\left(a/L\right)-16\left(M_{cr}/M_{a}-3\right){\left(a/L\right)}^{3}}{3\left(a/L\right)-4{\left(a/L\right)}^{3}}$$

Equation (13) for computing *I_e_* is provided using SIMTReC [8].

(13) $$I_{e-SIMTReC}=\frac{I_{un}I_{cr}}{I_{cr}+\left[1-0.5{\left(\frac{M_{cr}}{M_{a}}\right)}^{2}\right]\left(I_{un}-I_{cr}\right)}$$

According to ACI 318-19 [34], Equation (14) is a modified form of the Bischoff Equation created by introducing a reduced cracking moment. The effective cracking moment is taken as two thirds of the original cracking moment to account for the effects of the restraint conditions and reduced tensile strength of concrete.

(14) $$I_{e-ACI\;318}=\frac{I_{cr}}{1-{\left(\frac{\left(2/3\right)M_{cr}}{M_{a}}\right)}^{2}\left(1-\frac{I_{cr}}{I_{un}}\right)}$$

#### 4.2.2. Comparison between Experimental *I_e_* and Predictions

**Figure 12:**
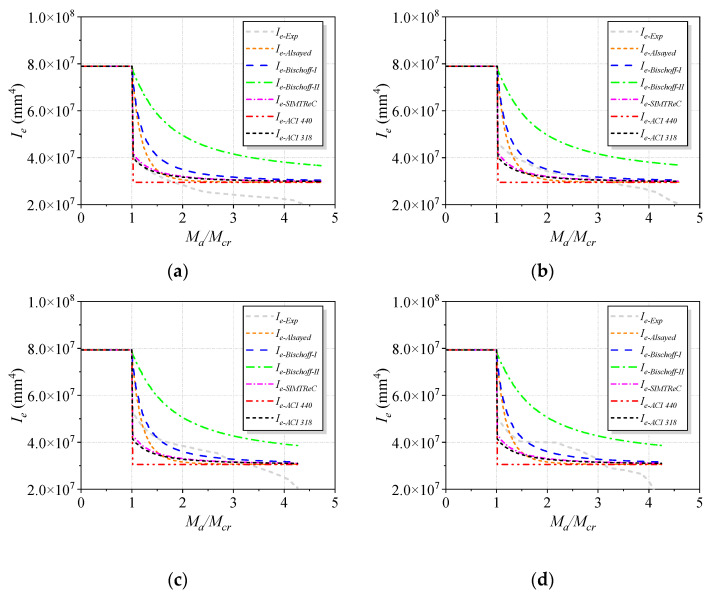

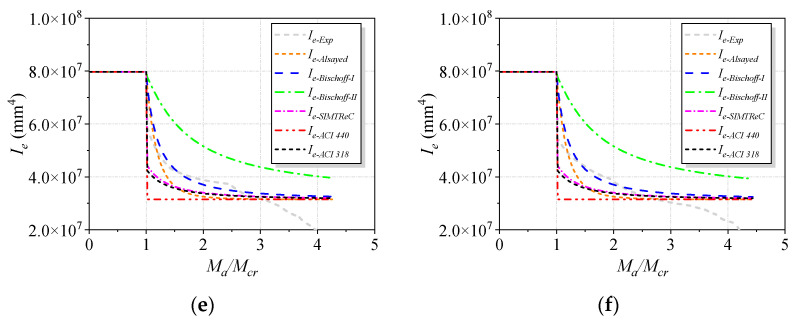
Comparisons between the experimental *I_e_* values and predictions for tested beams. (**a**) B0. (**b**) B1. (**c**) B3. (**d**) B3E. (**e**) B5. (**f**) B5E.

The experimentally evaluated *I_e_* values, according to Equation (8), were plotted versus those applied to the experimental cracking moment ratio (*M_a_/M_cr_)* for tested beams, as shown in Figure 12. *I_e_* predictions according to Alasyed et al., Bischoff-I, Bischoff-II, SIMTReC, ACI 440-15, and ACI 318-19 are also included in Figure 12 and were compared with the experimental values of *I_e_*. The Bischoff-II equation provided the largest overprediction of *I_e_*, and the defection was significantly underestimated as a result. The Bischoff-II Equation tended to lead to a higher *I_e_* than the Bischoff-I Equation, as the former incorporated a lower cracking moment, as observed in [33].

For B0, the SIMTReC and ACI 318-19 equations could effectively model the experimental *I_e_* immediately above the cracking moment. When *M_a_/M_cr_* increased to around 1.7, *I_e-Exp_* was overestimated by all the prediction equations. In addition, the Bischoff-I and Bischoff-II Equations provided an overestimate of *I_e_* but seemingly gave nearly identical overall tendencies. 

Regarding strengthened beams, the Alasyed et al., SIMTReC, and ACI 318-19 equations led to conservative predictions of *I_e_* after the cracking moment, and the differences between the experimental results and predictions decreased with increasing *M_a_/M_cr_* values. On the contrary, the Bischoff-II Equation significantly overestimated *I_e_* and led to a much smaller deflection value. It was found that the Bischoff-I Equation provided the prediction of *I_e_* with the best accuracy compared to the other equations, and this equation is recommended for computing the *I_e_* of strengthened beams. 

**Figure 13:**
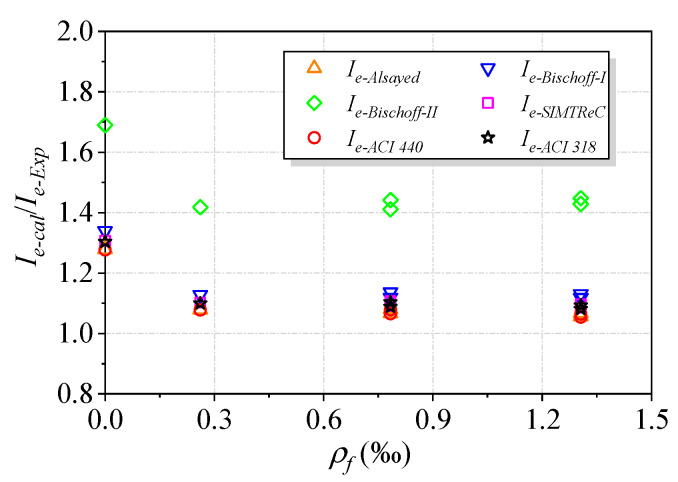
Comparison between the experimental *I_e_* values and predictions at a deflection limit of Δ/*L* = 360.

The purpose of predicting *I_e_* is to calculate the deflection to check whether the serviceability requirements related to deflection limits can be met for concrete beams in service conditions. For instance, ACI 318-19 provides a deflection limit for concrete beams, which is set as Δ/*L* = 360. The experimental *I_e_* and the predicted values corresponding to the above deflection limit were plotted with the reinforcement ratio of basal textile, as shown in Figure 13. Except for the Bischoff-II Equation, the overall ratios of the predicted to experimental *I_e_* values ranged between 1.06 and 1.13. The reason for the close predictions from different equations is that the deflection limit (Δ/*L* = 360) led to an *M_a_/M_cr_* value of about 3.5, at which the predicted *I_e_* curves were close to the experimental curve for all equations, as shown in Figure 12. Note that if the deflection limit was more restrictive (smaller deflections are permitted), the Bischoff-I Equation could still provide an accurate estimate of *I_e_*, while the predictions from other equations would significantly deviate from the experimental *I_e_*. Furthermore, future studies are still needed to estimate *I_e_* values for more relaxed deflection limits, as none of the existing models can accurately predict *I_e_* values for large *M_a_/M_cr_* values.

## 5. Conclusions

This study addressed the ultimate and deflection behavior of concrete beams strengthened in flexure with BTRM. The bond performance between the BTRM and concrete was investigated via single shear tests, and the effectiveness of using an adhesion promoter and impregnated resin on bond enhancement was evaluated. Furthermore, bending tests were conducted to study the flexural behavior of strengthened concrete beams in terms of failure modes, ultimate strength, and the effective moment of inertia. The following conclusions can be drawn based on the test results and analyses.

The bond failure between the BTRM and concrete occurred due to the debonding of BTRM. Using an adhesion promoter could shift the debonding failure to the tensile failure of the basalt textile, and textile tensile strength could be achieved. Utilizing both an adhesion promoter and impregnated resin led to textile failure with higher tensile strength, which was associated with the consolidating effects of the impregnated resin on the textile. In addition, the critical bond length required to achieve textile failure was equal to 125 mm when using the adhesion promoter and impregnated resin.

The use of BTRM significantly affected the flexural performance of strengthened beams. The strengthened beams with multiple textile layers failed due to end debonding at the interface between the textile and mortar. With the use of end anchorage, the failure mode changed to flexural failure, along with the tensile failure of the textile. The failure force of the beams increased with the reinforcement amount of basalt textile in the BTRM. The maximum increase in the failure force was 37%, which was achieved by the beam strengthened with five layers of the textile and the use of end anchorage.

A rectangular stress block approach was applied to calculate the moment capacity of BTRM-strengthened concrete beams. The calculated values were compared with the experimental results. The differences in the moment capacities between the calculations and test results were no more than 7%.

The deflection behavior could be indicated by an elastic deformation approach that incorporated the effective moment of inertia (*I_e_)*. The experimental value of *I_e_* was calculated using the load response and compared with different equations, including the Alasyed et al., Bischoff-I, Bischoff-II, SIMTReC, ACI 440-15, and ACI 318-19 equations. Among these equations, the Bischoff-I Equation provided the best approximation of *I_e_* compared to other prediction equations and thus is recommended for deflection behavior analysis for BTRM-strengthened beams when *M_cr_/M_a_* is no more than 3.0. 

## Figures and Tables

**Table 1:** Specimen arrangement and test results for bending tests.

ID	Textile Layers	End Anchorage	Textile Amount (‰)	Failure Load (kN)	Failure Mode
B0	0	No	0.00	93.5	CC
B1	1	No	0.26	105.5	CC+TF
B3	3	No	0.78	107.0	CC+EP
B3E	3	Yes	0.78	110.0	CC+TF
B5	5	No	1.31	122.1	SF+EP
B5E	5	Yes	1.31	128.1	SS+TF

Note: CC, TF, EP, and SF indicate concrete crushing, textile failure, end peeling, and shear failure, respectively.

**Table 2:** Comparison between the experimental and calculated moment capacity values.

ID	*M_u-Exp_* (kN·mm)	*M_u-Cal_* (kN·mm)	*M_u-Cal_/M_u-Exp_*
B0	17,523.9	17,080.9	0.97
B1	19,775.8	18,326.6	0.93
B3	20,063.1	---	---
B3E	20,628.2	20,598.9	1.00
B5	22,890.1	---	---
B5E	24,022.7	22,697.0	0.94
Average	---	---	0.96
COV	---	---	0.033

## Data Availability

Data will be made available on request.

## Notation

A_f_, A_s_, and A_s′_	the cross-sectional areas of basalt textile, tension steel, and compression steel;
a	the shear span of tested beams;
$d_{f},\;d_{s},\;\mathrm{and}\;d_{s}^{\prime }$	the distances between the extreme compression fiber of concrete and the centroid of basalt textile, tension steel, and compression steel;
E_c_, E_f_, and E_s_	the elastic moduli of concrete, basalt textile, and steel reinforcement;
I_cr_	the effective moment of inertia for a cracked section;
I_e_	the effective moment of inertia;
I_un_	the effective moment of inertia for an uncracked section;
L	the span of tested beams;
M_a_	the applied moment of tested beams;
M_cr_	the cracking moment of tested beams;
P_u_	the maximum applied force of tested beams obtained from bending tests;
x_c_	the depth of the neutral axis of the cracked section;
α_1_	the ratio of the block stress to the compressive strength of concrete;
β_1_	the ratio of the stress block height to the neutral axis depth of the cracked section;
Δ	the deflection of a tested beam at the midspan;
$\varepsilon_{c}^{\prime }$	the strain of concrete corresponding to the compressive strength;
ε_ct_	the strain at the top compression fiber of the cross-section of tested beams;
ε_f_	the strain in the basalt textile of tested beams;
ε_s_	the strain in the tension steel of tested beams;
$\varepsilon_{s}^{\prime }$	the strain in the compression steel of tested beams.
